# Cross‐sectional relations of whole‐blood miRNA expression levels and hand grip strength in a community sample

**DOI:** 10.1111/acel.12622

**Published:** 2017-06-08

**Authors:** Joanne M. Murabito, Jian Rong, Kathryn L. Lunetta, Tianxiao Huan, Honghuang Lin, Qiang Zhao, Jane E. Freedman, Kahraman Tanriverdi, Daniel Levy, Martin G. Larson

**Affiliations:** ^1^ The Framingham Heart Study Framingham MA USA; ^2^ Department of Medicine, Section of General Internal Medicine Boston University School of Medicine Boston MA USA; ^3^ Department of Biostatistics Boston University School of Public Health Boston MA USA; ^4^ The Population Sciences Branch Division of Intramural Research, National Heart, Lung, and Blood Institute National Institutes of Health Bethesda MD USA; ^5^ Section of Computational Biomedicine Department of Medicine Boston University School of Medicine Boston MA USA; ^6^ Cardiology Division Department of Medicine University of Massachusetts Medical School Worcester MA USA

**Keywords:** aging, epidemiology, grip strength, microRNA, mRNA

## Abstract

MicroRNAs (miRNAs) regulate gene expression with emerging data suggesting miRNAs play a role in skeletal muscle biology. We sought to examine the association of miRNAs with grip strength in a community‐based sample. Framingham Heart Study Offspring and Generation 3 participants (*n* = 5668 54% women, mean age 55 years, range 24, 90 years) underwent grip strength measurement and miRNA profiling using whole blood from fasting morning samples. Linear mixed‐effects regression modeling of grip strength (kg) versus continuous miRNA ‘Cq’ values and versus binary miRNA expression was performed. We conducted an integrative miRNA–mRNA coexpression analysis and examined the enrichment of biologic pathways for the top miRNAs associated with grip strength. Grip strength was lower in women than in men and declined with age with a mean 44.7 (10.0) kg in men and 26.5 (6.3) kg in women. Among 299 miRNAs interrogated for association with grip strength, 93 (31%) had FDR *q* value < 0.05, 54 (18%) had an FDR *q* value < 0.01, and 15 (5%) had FDR *q* value < 0.001. For almost all miRNA–grip strength associations, increasing miRNA concentration is associated with increasing grip strength. miR‐20a‐5p (FDR q 1.8 × 10^−6^) had the most significant association and several among the top 15 miRNAs had links to skeletal muscle including miR‐126‐3p, miR‐30a‐5p, and miR‐30d‐5p. The top associated biologic pathways included metabolism, chemokine signaling, and ubiquitin‐mediated proteolysis. Our comprehensive assessment in a community‐based sample of miRNAs in blood associated with grip strength provides a framework to further our understanding of the biology of muscle strength.

## Introduction

Hand grip strength is a simple and inexpensive measure of muscle strength associated with exceptional survival (Willcox *et al*., 2006), all‐cause mortality, cardiovascular mortality, and cardiovascular disease (Leong *et al*., 2015). Reduced grip strength is also associated with impaired mobility (Sallinen *et al*., 2010) and risk for physical disability (Cummings *et al*., 2014). Grip strength declines with age in both men and women (Dodds *et al*., 2014) and is used as a criterion of frailty as well as to define sarcopenia (low muscle mass and weakness; Studenski *et al*., 2014), which are important causes of disability and death in the community (Rodriguez‐Manas & Fried, 2014). There is interest in this measure as a phenotype of healthy aging and potentially to identify older adults for interventions to promote healthy aging (Studenski *et al*., 2014).

Biologic mechanisms influencing muscle strength maintenance and decline are complex with many contributing factors (Gonzalez‐Freire *et al*., 2014). Physical inactivity, malnutrition, hormonal changes, inflammatory pathway activation, mitochondrial dysfunction, obesity, glycemia, and age‐related diseases play a role in age‐related loss of muscle (Kalyani *et al*., 2014). In addition, genetic factors contribute substantially to the variability in grip strength (Frederiksen *et al*., 2002), suggesting that genetic studies may identify biologically relevant pathways important to muscle strength and aging. However, few genomewide association studies have been conducted of grip strength (Chan *et al*., 2015; Matteini *et al*., 2016) with one identifying a promising common variant in a chromosomal region linked to muscle repair (Matteini *et al*., 2016).

MicroRNAs (miRNAs) are small noncoding RNAs that regulate gene expression by altering mRNA transcripts. Differential expression of miRNAs has been associated with age (Noren *et al*., 2010, 2013) and age‐related diseases such as cancer and cardiovascular disease (Freedman *et al*., 2012). Emerging data suggest miRNAs may play a role in skeletal muscle biology including skeletal muscle development, muscle physiology, and specific muscle diseases (Goljanek‐Whysall *et al*., 2012; Kirby & McCarthy, 2013; Nie *et al*., 2015). Skeletal muscle‐enriched miRNAs have been identified in the circulation of both healthy individuals and those with muscle disease (Alexander & Kunkel, 2015; Denham & Prestes, 2016). In a small study of endurance athletes compared to healthy controls, muscle‐enriched circulating miRNAs with distinct roles in muscle biology measured in whole blood predict cardiopulmonary fitness parameters and change in abundance in response to a single bout of maximum aerobic exercise (Denham & Prestes, 2016). However, much of the work has been done in animal models and the molecular mechanisms of most differentially expressed miRNAs in skeletal muscle remain unknown. We had the opportunity to examine the association of circulating miRNAs with hand grip strength in a community‐based sample of adults across a wide age range.

## Results

**Figure 1:**
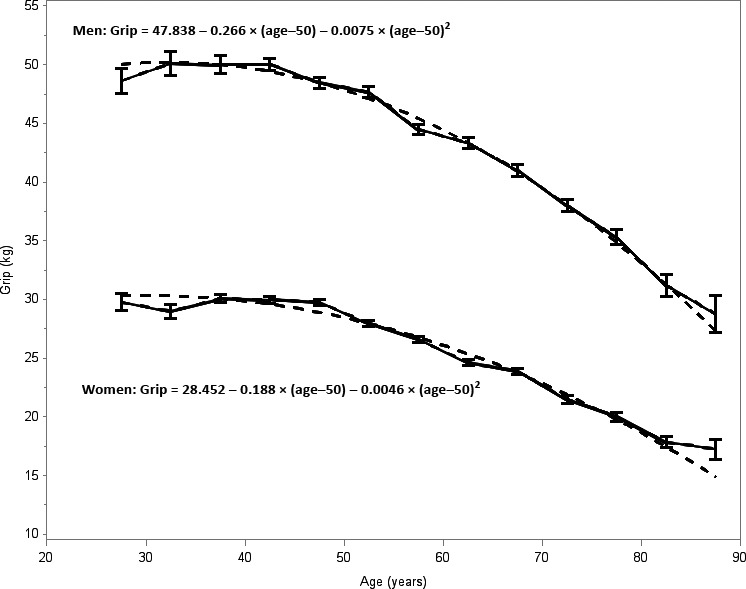
Hand grip strength (kg) by age groups in men and women. Plot shows mean and standard error bars for 5‐year age groups by sex. Men (mean 44.6 kg, SD 10.2); women (mean 26.2 kg, SD 6.5). A fitted regression with a quadratic age term in hashed lines estimates the rate of decline in grip strength.

Our investigation focused on Framingham Heart Study (FHS) Offspring participants who attended examination cycle 8 (2005–2008, *n* = 3021) and Gen 3 participants who attended examination cycle 2 (2008–2011, *n* = 3411), underwent hand grip strength testing, and had a blood sample for miRNA expression profiling. The characteristics of the 5668 Offspring and Gen 3 participants (54% women, mean age 55 years, range 24, 90 years) in the study sample are shown in Table 1 (Table S1, Supporting information provides characteristics by cohort). Hand grip strength was lower in women than in men and declined with age with a mean 44.7 (10.0) kg in men and 26.5 (6.3) kg in women (Fig. 1).

**Table 1:** Characteristics of the study sample

Characteristic	Overall (*N* = 5668)	Men (*N* = 2616)	Women (*N* = 3052)
Age, years	55.7 (13.2)	55.7 (13.1)	55.7 (13.2)
Women %	54	–	–
Grip Strength, kg	34.9 (12.3)	44.7 (10.0)	26.5 (6.3)
Height, inches	66.4 (3.75)	69.3 (2.67)	63.9 (2.5)
BMI, kg m^−2^	28.1 (5.6)	29.0 (4.9)	27.3 (6.1)
Current smoking, %	10	11	10
Hypertension, %	41	46	37
Diabetes, %	8.8	11.3	6.7
Total cholesterol, mg dL^−1^	186 (36)	181 (36)	191 (35)
HDL cholesterol. mg dL^−1^	59 (18)	50 (14)	66 (18)
Triglyceride, mg dL^−1^	115 (76)	127 (92)	105 (57)
Physical activity index	36 (6.1)	37 (7.3)	35 (4.9)
Cardiovascular disease, %	8.2	10.8	6.0

Data are presented as mean (SD) or percentage.

Linear mixed‐effects regression modeling of grip strength (kg) versus continuous miRNA ‘Cq’ values and also versus binary miRNA expression for miRNAs with measurable expression in at least 5% of the sample was conducted (see Experimental procedures for details). Among 299 miRNAs interrogated for association with hand grip strength, 112 (37%) had FDR *q* value < 0.10, 93 (31%) had FDR *q* value < 0.05, 54 (18%) had an FDR *q* value < 0.01, and 15 (5%) had FDR *q* value < 0.001 in models adjusted for sex, age, height, BMI, and technical variables (Table 2, Table S2, Supporting information). With further adjustment for cell counts (Table S3, Supporting information), we observed largely similar results. Among the top 15 associations in the primary analysis (Table 2), two‐thirds of miRNAs remained among the top 15 associations after adjusting for cell counts. The majority of the associations shown in Table 2 are derived from the continuous model (miRNA Cq values) with the exceptions of miR‐26a‐1‐3p where the association is observed in the binary model (miRNA expression yes/no) and miR‐668, miR‐25‐5p, and miR‐1304‐5p where the association is observed in both models. For almost all miRNA–hand grip associations, increasing miRNA concentration is associated with increasing grip strength. The direction of effect is consistent for the two models (continuous Cq values, binary expression yes/no) for most miRNAs. We examined the miRNAs expressed in > 90% of samples separately in men and women. There were 70 miRNAs in women and 48 miRNAs in men associated with hand grip at *P < *0.05 (Table S4, Supporting information). Among the top 15 miRNA associations, a greater number were seen among the top results in men than in women and the magnitude of most estimates was larger in men; however, the direction of the effect estimates were similar in men and women (Table S4, Supporting information).

**Table 2:** Top 15 miRNA associations with hand grip strength: Framingham Heart Study

miRNA	*N*	Linear regression, continuous miRNA: Cq values			Linear regression, binary miRNA expression (yes/no)			FDR q^a^
		Est Beta	SE	*P*‐value	Est Beta	SE	*P*‐value	
miR‐20a‐5p	5467	−0.20	0.03	6.03E‐09	−0.38	0.49	0.44	1.80E‐06
miR‐183‐3p	5492	−0.23	0.04	1.94E‐08	−0.65	0.53	0.22	2.90E‐06
miR‐29b‐2‐5p	5280	−0.32	0.06	5.78E‐08	−0.86	0.37	1.94E‐02	4.60E‐06
miR‐601	5542	−0.18	0.03	6.15E‐08	−1.54	0.63	1.46E‐02	4.60E‐06
miR‐766‐3p	5557	−0.29	0.05	8.23E‐08	−0.27	0.66	0.69	4.92E‐06
miR‐320b	5608	−0.33	0.06	1.73E‐07	0.64	0.89	0.47	7.43E‐06
miR‐942	5597	−0.30	0.06	1.74E‐07	−1.41	0.82	8.32E‐02	7.43E‐06
miR‐26a‐1‐3p	4820	−0.10	0.06	7.23E‐02	−1.33	0.27	8.69E‐07	3.85E‐05
miR‐30d‐5p	5604	−0.24	0.05	1.16E‐06	0.49	0.86	0.57	3.85E‐05
miR‐30a‐5p	5634	−0.25	0.05	3.62E‐06	−0.54	1.17	0.64	1.08E‐04
miR‐126‐3p	5621	−0.17	0.04	8.31E‐06	−0.84	0.995	0.40	2.25E‐04
miR‐668	4985	−0.12	0.04	1.91E‐03	−1.05	0.29	2.30E‐04	2.25E‐04
miR‐25‐5p	5389	−0.22	0.05	1.25E‐05	−1.42	0.43	9.18E‐04	2.88E‐04
miR‐18a‐5p‐a1	5432	−0.15	0.04	3.09E‐05	−0.95	0.46	3.96E‐02	6.60E‐04
miR‐1304‐5p	1469	0.14	0.04	3.46E‐04	−0.57	0.21	7.29E‐03	8.75E‐04

Models adjusted for age, sex, height, body mass index, technical covariates (RNA concentration, 260/280, RNA quality).

a Based on adaptive *P*‐values see statistical methods. Higher Cq values indicate lower miRNA expression levels. Therefore, negative beta values indicate positive associations between miRNA expression and grip strength.

Of 67 miRNAs reported to be associated with skeletal muscle development, muscle fiber physiology, or muscle disease in the published literature, nine were assayed and three were expressed in the FHS sample (Table S5, Supporting information). miR‐206 was associated with hand grip (FDR *q* = 0 = 0.012). Additionally, miR‐503 was expressed in 56 participants and associated with hand grip strength (FDR *q* = 0.002), however given the small sample results need to be interpreted with caution.

To better understand how the top 15 miRNAs associated with hand grip strength might contribute to muscle strength and healthy aging, we analyzed miRNA–mRNA coexpression in the same set of FHS participants (*n* = 5340) in whom miRNA and mRNA expression data were both available (Table S6, Supporting information). At an FDR < 0.05, there were 2895 miRNA–mRNA coexpressed pairs that ranged from 0 to 1 pair for a given miRNA (miR‐18a‐5p‐a1, miR‐1304‐5p, respectively) to 477 pairs (miR‐126‐3p). These pairs consisted of 1560 unique mRNA genes, which were considered as potential genes targets of 15 hand grip strength‐associated miRNAs. Among the 2895 pairs, 1394 pairs were also predicted by TargetScan. We also examined the 2895 miRNA–mRNA pairs for the presence of the 150 genes derived from muscle that denote a healthy aging gene signature (Sood *et al*., 2015). There were 31 miRNA–mRNA pairs among this healthy aging signature representing 17 unique genes. Next, we examined the 1560 unique genes from the coexpressed miRNA–mRNA pairs in WebGestalt to identify enrichment of biologic pathways. The top ten biologic pathways are consistent with pathways important not only with muscle function but with aging itself including metabolic, chemokine signaling, ubiquitin‐mediated proteolysis, and RNA transport among others (Table 3). Table S7 (Supporting information) lists the genes and associated miRNAs in each pathway. We also tested the enrichment of these potential gene targets among known aging‐related pathways, and found that both the insulin signaling pathway and mTOR pathway (gene list in Table S8, Supporting information) were significantly enriched (*P* = 3.58E‐6 and *P* = 6.52e‐5, respectively). Interestingly, the potential gene targets were also highly enriched (*P* < 1e‐16) with aging‐related genes identified previously (Peters *et al*., 2015). Finally, we conducted a miRNA–mRNA coexpression analysis for 11 miRNAs with a positive beta estimate (negative association with grip strength) among the 93 miRNA–grip associations with FDR < 0.05 in our primary analysis (Table S2, Supporting information) and conducted pathway analysis on the 920 unique associated genes. The top biologic pathways with FDR < 0.05 relate to immune function, chemokine signaling, and apoptosis among others (Table S9, Supporting information, pathways, genes, and associated miRNAs for each pathway). Many of the 11 miRNAs come from the present/absent model and are not significant in the model with continuous Cq expression values (model restricted to participants with detectable levels of the miRNA) and four of the 11 miRNAs are available in a sample of under 1000 individuals.

**Table 3:** Top biologic pathways based on miRNA–mRNA coexpression pairs from top 15 miRNAs and the unique 1500 associated genes

Pathway name	Genes in pathway	Ratio of enrichment	*P*‐value	FDR Q value
Metabolic pathways	102	2.60	2.0E‐18	3.5E‐16
Chemokine signaling pathway	36	5.49	7.5E‐17	6.5E‐15
Regulation of actin cytoskeleton	37	5.01	6.4E‐16	2.8E‐14
Endocytosis	36	5.16	5.8E‐16	2.8E‐14
Ubiquitin‐mediated proteolysis	28	5.98	2.1E‐14	7.3E‐13
Huntington's disease	32	5.04	4.8E‐14	1.4E‐12
Alzheimer's disease	30	5.18	1.4E‐13	3.5E‐12
RNA transport	27	5.15	2.6E‐12	5.6E‐11
Protein processing in endoplasmic reticulum	27	4.72	2.2E‐11	4.3E‐10
Spliceosome	23	5.22	8.1E‐11	1.4E‐09

## Discussion

In the present study, we examined the relation of 299 miRNAs measured in whole blood and hand grip strength in a community‐based sample of men and women across a wide age range. In our sample, 93 miRNAs were associated with hand grip strength (FDR *q* < 0.05). For the top 15 miRNAs, increasing miRNA concentration was associated with increasing grip strength for nearly all associations. Using an integrative miRNA–mRNA coexpression analysis, we identified the potential gene targets (*N* = 1560 unique genes) for the top 15 hand grip strength‐associated miRNAs. Consistent with our hypothesis that grip strength is a healthy aging phenotype, the top biologic pathways were important to aging itself and the potential gene targets were enriched for previously identified age‐related genes.(Peters *et al*., 2015).

miR‐20a‐5p, the strongest miRNA associated with hand grip strength in our study, is a member of the miR‐17 family known to have many biologic functions including a role in hepatic insulin resistance (Fang *et al*., 2016). Its function in muscle is not well studied nor completely understood; however, recent work suggests a potential role as a biomarker for osteosarcoma (Li *et al*., 2015). Several miRNAs among the top 15 hand grip strength‐associated miRNAs have links to skeletal muscle. A small study of healthy young and old men demonstrated that miR‐126 was downregulated after resistance exercise in older men but not young men (Rivas *et al*., 2014). Follow‐up mechanistic work revealed that decreased miR‐126 in proliferating myoblasts impacts the IGF‐1 signaling pathway, an important regulator of skeletal muscle growth. This noted impairment in exercise‐induced miRNA regulation with aging and the associated inhibition of the IGF‐1 signaling may provide a mechanism for age‐related muscle loss such as sarcopenia (Rivas *et al*., 2014). The miR‐30‐5p family is known to play a role in cardiomyocyte and vascular smooth muscle cell pathophysiology (Balderman *et al*., 2012; Yin *et al*., 2013). In animal models, the miR‐30 family (including miR‐30a‐5p and miR‐30d‐5p) regulates differentiation of myoblasts and may be a biomarker of disturbed muscle homeostasis (Guess *et al*., 2015). Recent work in bovine models supports a role for miR‐30‐5p in skeletal muscle development and regulation of alternative splicing of key muscle genes by targeting the MBNL (muscleblind‐like) family (Zhang *et al*., 2016).

miRNA analysis conducted on skeletal muscle biopsies from young and older adults identified the Let‐7 family, significantly Let‐7b and Let‐7e, as having higher expression in older adults (Drummond *et al*., 2011). All mean let‐7 levels for the 10 family members assayed in that study were higher in older subjects raising the possibility that the Let‐7 family members work together to alter gene expression or that with further aging additional Let‐7 family members would become significant (the sample size was small). In our study, several Let‐7 family members assayed in blood were associated with hand grip strength (Let‐7c FDR *q* = 0.005, Let‐7g‐5p FDR *q* = 0.004, Let‐7e‐5 FDR *q* = 0.013, Let‐7a‐5p FDR *q* = 0.03). The Let‐7 family is important in tumor suppression and cell cycle control. It is possible that increased expression of Let‐7 with aging impairs cellular proliferation and muscle regeneration capacity (Drummond *et al*., 2011).

We looked up the miRNAs reported to be associated with skeletal muscle development, muscle fiber physiology, or muscle disease in the published literature in our study results and found that miR‐206 was significantly associated with hand grip strength (FDR *q* = 0.012). miR‐206 is a muscle‐enriched miRNA known as myomiRs and has been associated with a number of age‐associated changes in muscle pathophysiology including changes in type II muscle fibers, reduction in neuromuscular junction signaling, and decreases in muscle satellite cell numbers (Brown & Goljanek‐Whysall, 2015; Nie *et al*., 2015). In studies of young and old mice, expression of miR‐206 has been associated with age‐related muscle atrophy which may provide insights into the human age‐related condition of sarcopenia (Kim *et al*., 2014; Nie *et al*., 2015).

Participants in our sample had both miRNA and mRNA data permitting a coexpression analysis of the top 15 miRNAs associated with grip strength and a further examination of the potential unique gene targets of the miRNAs. The genes were enriched for age‐related genes (Peters *et al*., 2015) and biologic pathways important in muscle physiology including among the top ten pathways metabolism, chemokine signaling, RNA transport, and ubiquitin‐mediated proteolysis (Lopez‐Otin *et al*., 2013). The ubiquitin/proteasome pathway (UPP) plays a crucial role in muscle health including muscle cell differentiation, muscle growth and integrity, and muscle mass maintenance (Bell *et al*., 2016). There were many ubiquitin genes (Table S6, Supporting information) in our pathway analysis associated with the top 15 hand grip‐associated miRNAs. It is of interest that the genes associated with the downregulated miRNAs from the miRNA–mRNA coexpression function in biologic pathways linked to immune function and inflammation given the key role this pathway plays in muscle strength decline (Gonzalez‐Freire *et al*., 2014) and aging.

Our study had several strengths including the number of miRNAs profiled, the large sample size and broad age range of participants, and the community‐based setting unselected for any specific disease. The study also has limitations including the lack of race/ethnic diversity limiting generalizability of findings beyond participants of white, European ancestry background. The miRNAs were assayed in blood as our study is an epidemiologic cohort study without accessibility to muscle tissue; however, several of our findings are consistent with small studies (*n *< 50) of miRNAs profiled in skeletal muscle biopsies. Finally, our findings need to be replicated in an independent sample and further functional studies to determine the underlying biologic mechanisms of the miRNA–grip strength associations are needed.

In conclusion, in our community‐based sample of men and women across a wide age range, we identified 93 miRNAs associated with hand grip strength. The potential gene targets for the top associations are linked to biologic pathways important to muscle and aging including metabolism, chemokine signaling, and ubiquitin‐mediated proteolysis. These results need to be replicated in an independent human sample but provide a framework to further our understanding of the biology of muscle strength and healthy aging phenotypes.

## Experimental procedures

### Study sample

The Framingham Heart Study (FHS) is an ongoing prospective community‐based family study that includes the Offspring cohort enrolled in 1971 and the children of the Offspring participants who were enrolled in the Third Generation cohort (Gen 3) in 2002 (Splansky *et al*., 2007). For the present investigation, we focused on Offspring participants who attended examination cycle 8 (2005–2008, *n* = 3021) and Gen 3 participants who attended examination cycle 2 (2008–2011, *n* = 3411), underwent hand grip strength testing, and had a blood sample for miRNA expression profiling. We excluded 108 participants with missing grip strength data, 638 participants without miRNA expression data, and 18 participants with missing covariate data providing a study sample of 5668 participants. All participants provided informed consent and the examination protocols were approved by the Boston University Medical Center Institutional Review Board.

### Hand grip measurement

Hand grip strength was obtained by trained technicians with a JAMAR dynamometer (Model #5030J1, Sammons Preston/JLW Instruments, Chicago, IL, USA) using the same protocol for both Offspring and Gen 3 participants. The participant was seated with forearm resting on the arm of a chair and instructed to hold the dynamometer upright and squeeze as hard as able. Three trials in the right hand and then the left hand were recorded. The maximum of the six trials was used for analysis.

### miRNA expression profiling

Whole blood from fasting morning samples were used for miRNA profiling. The high‐throughput Gene Expression Core Laboratory at the University of Massachusetts Medical School profiled all TaqMan miRNA commercially available assays (*n* = 774) available at the start of the Systems Approach to Biomarker Research in Cardiovascular Disease (SABRe CVD) Project (McManus *et al*., 2017). If the miRNA was not detected in a sample of 550 participants, it was removed from further study and not measured in the full cohort. The remaining miRNA profiling in FHS was carried out on 346 miRNAs using quantitative real‐time polymerase chain reaction (RT‐qPCR) using TaqMan chemistry‐based miRNA assays. The qPCRs were conducted using a high‐throughput instrument (BioMark, Fluidigm, San Francisco, CA, USA). In reproducibility analysis, for replicates > 95% of data points had coefficients of variation < 10%. miRNA expression is quantified using cycle threshold (Cq) where lower Cq values represent higher miRNA expression levels. Low expression using the nano‐volume Fluidigm system is considered as a Cq value of 27 or higher. We evaluated 299 miRNAs with measurable Cq values in at least 5% (250) of participants.

### Gene expression (mRNA)

Gene expression profiling was performed from blood samples taken on Offspring examination 8 (*n* = 2442) and Gen 3 examination 2 (*n* = 3180) as previously described (Joehanes *et al*., 2013). In brief, the Affymetrix Human Exon 1.0 ST Array (Affymetrix, Inc, Santa Clara, CA) was used and gene annotations were obtained from Affymetrix NetAffx Analysis Center (version 31) resulting in 17,324 distinct genes for downstream analysis.

### Covariates

At each FHS examination, height and weight were obtained according to standardized protocols and body mass index was calculated as weight in kilograms divided by the square of the height in meters (kg m^−2^). Resting blood pressure was obtained twice by the physician and hypertension was considered present if the average blood pressure measurement was ≥ 140/90 or the participant reported antihypertensive medications. Fasting laboratory measurements include glucose and lipids. Diabetes was defined as a fasting plasma glucose > 125 mg dL^−1^ or treatment with medications. Current smokers were defined as smoking 1 or more cigarettes per day during the year prior to the examination. Participants reported the number of hours per day spent in sleep, sedentary, slight, moderate, and heavy activities to calculate the physical activity index. Prevalent cardiovascular disease was defined as coronary heart disease, stroke, or intermittent claudication using previously established criteria.

### Statistical analysis

Many participants did not express some miRNAs at detectable levels. Therefore, for each miRNA in each participant, we defined a binary variable to code whether the miRNA was not detectable: *X* = 1 if Cq > 27, 0 otherwise. Thus, *X* = 1 corresponds with low (undetectable) concentration, whereas *X* = 0 corresponds with detectable miRNA concentration. We fitted two linear mixed‐effects regression models for each miRNA: (model 1) in everyone, grip ~ X and covariates, and (model 2) in the subset having *X* = 0, grip ~ Cq and covariates. We combined results from these two analyses as follows. If an miRNA was expressed in < 10% of samples, we used only model 1; if it was expressed in at least 90% of samples, we used only model 2; if it was expressed in 10% to < 90% of samples, we added chi‐squared statistics from both models and calculated a *P*‐value from a chi‐squared distribution with two degrees of freedom. Our primary analysis adjusted for sex, age, height, BMI, and technical variables (RNA concentration, RNA quality, and RNA 260/280 ratio). For miRNAs expressed in at least 90% of samples, we examined associations with grip strength separately in women and men to assess whether the relations were similar in both sexes.

In secondary analyses, we also adjusted for cell counts: red blood cell count, white blood cell count, platelets, percent neutrophil, percent lymphocytes, percent monocytes, and percent eosinophils. [Blood cell counts were measured in Gen 3 participants and partial least‐squares regression was used to impute cell counts with 10‐fold cross‐validation (Pilling *et al*., 2015)]. Imputed cell counts were used in these analyses.

To evaluate our approach, we conducted permutation testing by separating miRNA data from clinical data (hand grip and covariates: age, sex, BMI, RNA quality, concentration, 260/280 ratio, and cell count variables). We randomly ordered the clinical data, merged that with miRNA data, and fitted the two covariate‐adjusted models for grip versus (i) binary and ((ii) continuous miRNA. From each pair of models, we calculated *P*‐values using the adaptive test. We ran 100 replicates to examine distributions of adaptive *P*‐values and we established that type I error was not inflated.

Following regression analyses, we examined miRNAs reported in the literature to be associated with skeletal muscle development, muscle fiber physiology, or muscle disease such as sarcopenia in humans or animal models (Goljanek‐Whysall *et al*., 2012; Smith‐Vikos & Slack, 2012; Kirby & McCarthy, 2013; McGregor *et al*., 2014; Rivas *et al*., 2014; Nie *et al*., 2015) to determine whether these miRNAs were assayed and expressed in our community sample of adults. Finally, we checked whether those expressed miRNAs were associated with hand grip strength.

### miRNA–mRNA coexpression and pathway analysis

We also performed a miRNA–mRNA coexpression analysis among Offspring and Gen 3 participants with both miRNA and mRNA data. Linear mixed‐effects models (R package *lmekin()* function) were used to conduct pairwise coexpression analyses for all profiled mRNAs (*N* = 17,318) and the top 15 miRNAs. Significant miRNA–mRNA coexpression pairs were selected using FDR < 0.05. TargetScan v7.0 (Lewis *et al*., 2005; Agarwal *et al*., 2015) was used to predict whether the mRNAs were the corresponding targets for the miRNAs for the coexpressed miRNA–mRNA pairs identified. Pathway analyses were conducted in DAVID Bioinformatics resources 6.7 (https://david.ncifcrf.gov/) (Huang *et al*., 2009), and WebGestalt (Wang *et al*., 2013) to identify significant biologic mechanisms associated with the unique genes that were associated with the top 15 miRNAs. In addition, we examined the enrichment of grip strength‐related genes in key aging pathways (insulin signaling/IGF1/FOXO and mTOR pathways) and among the genes associated with aging in a large human meta‐analysis (Peters *et al*., 2015). The key aging pathways have also been important in muscle physiology with insulin‐like growth factors important to muscle cell differentiation and muscle regeneration (Zanou & Gailly, 2013). Finally, we investigated whether the 150 genes comprising the RNA signature of healthy human aging derived from muscle tissue (Sood *et al*., 2015) were targets of the top 15 miRNAs associated with hand grip in this study.

We conducted a second miRNA–mRNA coexpression analysis for miRNAs that were negatively associated with grip strength. Among 93 miRNA–hand grip associations with FDR < 0.05 in our primary analysis (Table S2, Supporting information), we identified 11 miRNAs with a positive beta estimate (miR‐149‐5p, miR‐126‐5p, miR‐1249, miR‐193b‐3p, miR‐219‐1‐3p, miR‐1274A, miR‐1276, miR‐1179, miR‐99b‐3p, miR‐491‐5p, miR‐543). Four of the 11 miRNAs come from the present/absent model and are not significant in the model with continuous Cq expression values. Of note, four of the 11 miRNAs are available in a sample of under 1000 individuals. We ran miRNA–mRNA coexpression and conducted pathway analyses using the same methods described above.

## Funding

This analysis was funded through a grant from NIA, R56AG029451. The Framingham Heart Study is funded by National Institutes of Health contract N01‐HC‐25195 and HHSN268201500001I. The laboratory work for this investigation was funded by the Division of Intramural Research, National Heart, Lung, and Blood Institute, National Institutes of Health.

## Author contributions

JMM, MGL, and KLL designed and supervised the project. KT and JEF performed the microRNA profiling at the Gene Expression and Biomarker Core Laboratory at the University of Massachusetts Medical School. DL was the PI of the SABRe project. JR conducted the main analyses and MGL supervised the analyses. TH and HL performed the coexpression and bioinformatics analyses. JMM drafted the manuscript with all authors participating in revision of the manuscript. All authors reviewed and approved the final manuscript.

## Conflict of interest

None declared.

## Supporting information


**Table S1** Characteristics of the study sample by cohort.
**Table S2** Primary model miRNA associations with hand grip strength.
**Table S3** Secondary model: miRNA associations with hand grip strength, additional adjustment for cell counts.
**Table S4** Secondary model: miRNA associations with hand grip strength, additional adjustment for cell counts.
**Table S5** miRNAs associated with muscle in the literature.
**Table S6** (a) miRNA–mRNA coexpression analysis.
**Table S7** Pathway analysis top 15 miRNAs.
**Table S8** Gene list for custom pathways.
**Table S9** Pathway analysis 11 miRNAs negatively associated with grip strength.
